# Central and lateral neck involvement in papillary thyroid carcinoma patients with or without thyroid capsular invasion: A multi-center analysis

**DOI:** 10.3389/fendo.2023.1138085

**Published:** 2023-03-09

**Authors:** Zheyu Yang, Yu Heng, Jian Zhou, Lei Tao, Wei Cai

**Affiliations:** ^1^ Department of General Surgery, Ruijin Hospital, Shanghai Jiaotong University School of Medicine, Shanghai, China; ^2^ Department of Otorhinolaryngology, ENT Institute, Eye & ENT Hospital, Fudan University, Shanghai, China

**Keywords:** papillary thyroid carcinoma, central lymph node metastasis, lateral lymph node metastasis, thyroid capsular invasion, risk stratification

## Abstract

**Purposes:**

To quantitatively predict the probability of cervical lymph node metastasis for papillary thyroid carcinomas (PTC) patients with or without thyroid capsular invasion (TCI), to guide the decision-making of management strategies for neck regions.

**Methods:**

A total of 998 PTC patients from three medical centers were retrospectively analyzed.

**Results:**

Patients with positive TCI (TCI group) exhibited higher risks for both CLNM and LLNM than those with negative TCI (no-TCI group). Patients receiving lateral lymph node dissection showed significantly higher incidence of relatively severe postoperative complications. For no-TCI group, factors including age less than 55 years old, male, the presence of bilateral disease and multifocality, and maximum tumor diameter (MTD)>=0.5cm were confirmed to be independent risk factors for CLNM, while the presence of bilateral disease and ipsilateral nodular goiter (iNG), and maximum positive CLN diameter (MCLND)>1.0cm independent factors for LLNM. Independent risk factors of LLNM for patients within the TCI group included MCLND>1.0cm, positive CLN number>=3, and the presence of iNG. Predictive models of CLNM and LLNM were established based on the aforementioned risk factors for patients within no-TCI and TCI groups. A meticulous and comprehensive risk stratification flow chart was established for a more accurate evaluation of central neck involvement including both CLNM and LLNM risk in PTC patients.

**Conclusions:**

A meticulous and comprehensive stratification flow chart for PTC patients for quantitatively evaluating both CLNM and LLNM was constructed.

## 1 Introduction

The incidence of Papillary thyroid carcinoma (PTC) continues to increase in recent years, ranking first among all kinds of malignancy of the endocrine system (1, 2). Although PTC patients generally exhibit satisfactory long-term overall and recurrence-free survival, approximately 20%-80% of patients were found to have neck metastasis when diagnosed (3–5). Ultrasonography (US) is the most common detection technique for the diagnosis of PTC. Although the application of high-resolution ultrasonography (US) and US-guided fine-needle aspiration (FNA) biopsy have increased the detection rate of PTC and corresponding neck involvement, it has been reported that the primary imaging modality ultrasound showed low sensitivity for detecting neck involvement, especially for the central compartment, which may be shadowed by nearby tissues including thyroid, collarbone, and trachea (6, 7).

Previous studies have shown that the rate of undetected central lymph node metastasis (CLNM) before surgery was higher than 30% (8, 9), thus prophylactic central lymph node dissection (CLND) is strongly recommended for all patients with clinical N0 PTC in many clinical centers. However, prophylactic surgical intervention involving the central neck compartment is considered over-treatment in some regions. Therefore, the management of the central neck is still controversial for PTC patients (10). On the other hand, although the detection rate of preoperative US for lateral lymph node metastasis (LLNM) was relatively high, ranging from 60.5% to 80.3% (11, 12), the occult LLNM rates are still reported to hover at 20% to 40% (13, 14). Furthermore, given the relatively high risk of postoperative complications resulting from lateral lymph node dissection (LLND), prophylactic LLND is generally not accepted as a standard strategy for PTC patients. Thus, there is great value in developing a more refined stratification system for assessing LLNM risk in PTC patients.

Thyroid capsular invasion (TCI), which is defined as a tumor clinging close to the junction of the thyroid and adjacent soft tissue and has invaded beyond the thyroid and surrounding fibrous, fat, and even skeletal muscle tissues (13), has been proven to be significantly associated with both CLNM and LLNM (8, 13, 14). Patients with TCI exhibit significantly higher neck involvement rates compared to those with no TCI, indicating distinct local metastasis risks between patients with or without TCI. However, these two groups of patients are often analyzed together, and no existing literature has discussed those patients respectively.

Here in our study, a comprehensive and meticulous evaluating system that can efficaciously quantify risks of CLNM and LLNM for PTC patients with TCI or not was established.

## 2 Materials and methods

### 2.1 Patient cohort

The study has been submitted to the Chinese Clinical Trial Registry chictr.org.cn. The assigned Unique Identifying Number is ChiCTR2100043353.

Initial surgery was conducted for 1112 patients with thyroid cancer at the following three hospitals between 2018 and 2020: Department of Otorhinolaryngology, Head and Neck Surgery at the Eye, Ear, Nose and Throat Hospital of Fudan University, Department of General Surgery at Ruijin Hospital of Shanghai Jiao Tong University School of Medicine, and Department of General Surgery, Civil Aviation Shanghai Hospital. Patients with any of the following criteria were excluded from this research: 1) Pathological type other than PTC (N=73); 2) Having received thyroid-related surgery previously (N=27); 3) History or coexistence of other primary tumors (N=14). At last, a total of 998 patients were enrolled in this research. This study was approved by the Institutional Ethics Committee of the Eye & ENT Hospital of Fudan University, the Ruijin Hospital of Shanghai Jiao Tong University School of Medicine, and the Department of General Surgery, Civil Aviation Shanghai Hospital. All participants gave informed consent to take part in the study.

### 2.2 Surgical management and cervical lymph node metastases

Aside from a total thyroidectomy or thyroid lobectomy, CLND was prophylactically conducted for every patient. LLND was performed for patients with preoperative detected LLNM by US or US-guided FNA. Those highly suspected to have lateral neck metastases were assessed by surgeons before or during operation. Clinicopathological information including TCI, maximum tumor diameter (MTD), multifocality, CLNM, LLNM, positive CLN number (CLNN), maximum positive CLN diameter (MCLND), ipsilateral nodular goiter (iNG) and ipsilateral Hashimoto thyroiditis, were obtained from histopathological results of surgical specimens. The thyroid glands were categorized into three equal volumes (upper portion, middle portion, and lower portion) according to the generally accepted consensus. Tumors with a maximum diameter of more than 2.0cm, primarily located in the upper 1/3 portion, and do not exceed the lower 1/3 thyroid gland were also defined as upper portion tumors.

### 2.3 Statistical analysis

Statistical analysis was performed using SPSS Statistics version 24.0 (SPSS Inc., Chicago, IL, USA). Chi-square and independent t-tests were conducted respectively for the comparison of categorical and continuous variables between different groups. Logistic univariate and multivariate regression analyses were performed for screening out independent predictors for CLNM and LLNM for PTC patients, which were further used for constructing a nomogram. The construction of the nomogram was performed by R software (version 3.5.1; R Development Core Team). Then the concordance index (C-index), receiver operating characteristic (ROC) curve, and the calibration curve were used for examination of our newly created nomograms. Patients were divided into different subgroups with extremely different CLNM and LLNM neck involvement rates According to the distribution of total scores based on newly-created nomograms respectively. A P value of <0.05 was considered statistically significant.

## 3 Results

### 3.1 Patients’ basic demographics and clinicopathological characteristics

A total of 998 patients who were diagnosed as PTC by postoperative pathology were analyzed in this research. The mean age of all patients was 42.68 years, with an SD of 12.52 years. The baseline demographics and clinicopathological characteristics of the patients enrolled are shown in **Table 1**. Patients with negative TCI (No-TCI group) were significantly older than those with positive TCI (TCI group, 43.80± 12.38 vs. 40.83 ± 12.56, p-value= 0.000). Patients within the TCI group showed significantly larger tumor sizes than those within the no-TCI group, and the presence of bilateral disease, multifocality, and iNG were significantly more common in the TCI group than in the no-TCI group (p-value<0.05). However, ipsilateral Hashimoto thyroiditis was more common in the no-TCI group than in the TCI group (p-value=0.008).

**Table 1:** The clinicopathological characteristics of all patients.

	All Patients		No-TCI		TCI		
	n=998	%	n=622	%	n=376	%	P value
**Age (mean ± SD)**	42.68 ± 12.52		43.80 ± 12.38		40.83 ± 12.56		0.000
**BMI (mean ± SD)**	23.73 ± 3.76		23.66 ± 3.78		23.85 ± 3.74		0.458
**Maximum tumor diameter (mean ± SD)**	0.88 ± 0.72		0.74 ± 0.59		1.12 ± 0.83		0.000
**Gender**							0.022
Male	199	19.9	138	22.2	61	16.2	
Female	799	80.1	484	77.8	315	83.8	
**History of smoking**							0.354
No	928	93.0	582	93.6	346	92.0	
Yes	70	7.0	40	6.4	30	8.0	
**History of alcoholism**							0.381
No	930	**93.2**	583	93.7	347	92.3	
Yes	68	6.8	39	6.3	29	7.7	
**History of hypertension**							0.759
No	817	81.9	511	82.2	306	81.4	
Yes	181	18.1	111	17.8	70	18.6	
**History of diabetes**							0.086
No	927	92.9	571	91.8	356	94.7	
Yes	71	7.1	51	8.2	20	5.3	
**Bilateral disease**							0.005
Absent	792	79.4	511	82.2	281	74.7	
Present	206	20.6	111	17.8	95	25.3	
**Multifocality**							0.000
Absent	700	70.1	465	74.8	235	62.5	
Present	298	29.9	157	25.2	141	37.5	
**Tumor location**							0.097
Upper portion	270	27.1	157	25.2	113	30.1	
Middle/Lower portion	728	72.9	465	74.8	263	69.9	
**Number of positive CLN** **(for positive CLNM only, n =503)**							0.084
1-2	256	50.9	120	56.3	136	46.9	
3-4	117	23.3	47	22.1	70	24.1	
>= 5	130	25.8	46	21.6	84	29.0	
**Maximum diameter of positive CLN** **(for positive CLNM only, n =503)**							0.256
< 1.0cm	389	77.3	170	79.8	219	75.5	
>= 1.0cm	114	22.7	43	20.2	71	24.5	
**PTC with ipsilateral Hashimoto thyroiditis**							0.008
No	798	80.0	481	77.3	317	84.3	
Yes	200	20.0	141	22.7	59	15.7	
**PTC with ipsilateral nodular goiter**							0.001
No	712	71.3	467	75.1	245	65.2	
Yes	286	28.7	155	24.9	131	34.8	
**CLNM**							0.000
No	495	49.6	409	65.8	86	22.9	
Yes	503	50.4	213	34.2	290	77.1	
**LLNM** **(for positive CLNM only, n =503)**							0.001
No	383	76.1	178	83.6	205	70.7	
Yes	120	23.9	35	16.4	85	29.3	
**Recurrence**							0.039
No	963	96.5	606	97.4	357	94.9	
Yes	35	3.5	16	2.6	19	5.1	

TCI, thyroid capsular invasion; BMI, body mass index; CLN, central lymph node; PTC, papillary thyroid carcinoma; CLNM, central lymph node metastasis; LLNM, lateral lymph node metastasis.

As CLND was prophylactically performed for all patients regardless of whether central neck involvement was detected before surgery, CLNM was easy to confirm by postoperative pathology. 503 (50.4%) patients exhibited CLNM in our cohort, with 290 (77.1%) in the TCI group and 213 (34.2%) in the no-TCI group. Patients with positive TCI were significantly more likely to develop central neck involvement than those with negative TCI (p-value=0.000).

**Figure 1:**
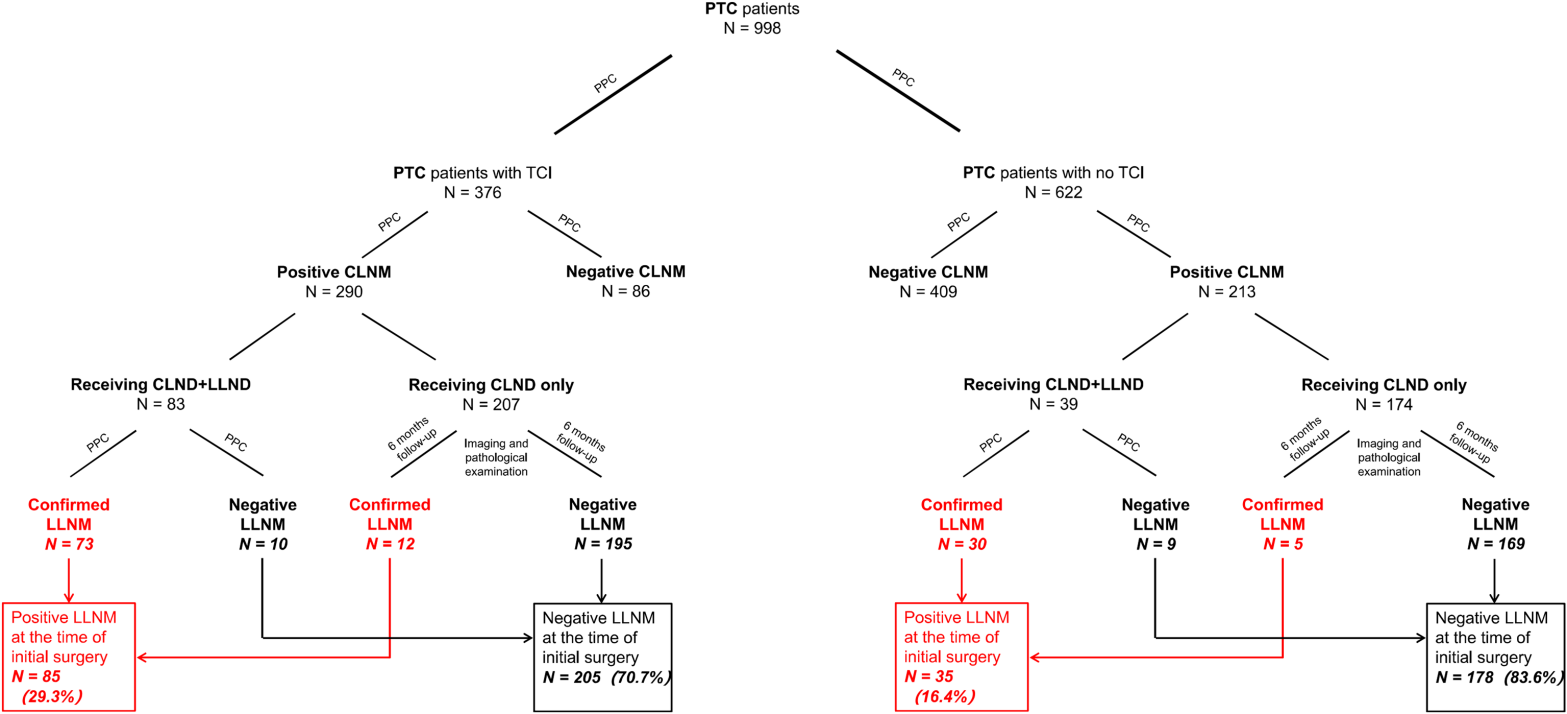
Flow diagram of case selection steps and follow-up information for patients enrolled. PTC, papillary thyroid carcinoma; PPC, postoperative pathology confirmed metastasis; CLNM, central lymph node metastasis; LLNM, lateral lymph node metastasis; CLND, central lymph node dissection; LLND, lateral lymph node dissection.

However, LLND was conducted only for those with preoperatively detected positive or highly suspicious LLNM by surgeons before or during intraoperative phases. In the TCI group, 83 of the 290 patients with positive CLNM received CLND+LLND and 73 patients were diagnosed as having lateral neck involvement by postoperative pathology. 12 of the other 207 patients with positive CLNM receiving CLND alone in the TCI group were detected to have LLNM within six months after the initial surgery. In total, 85 (29.3%) of 290 patients with positive CLNM in the TCI group were considered as having lateral lymph node involvement before surgery (Diagram shown in **Figure 1**). For the 213 patients with positive CLNM within the no-TCI group, 39 patients received LLND and 30 of them were proven to have pathological-detected CLNM. Among the other 174 patients receiving CLND only, 5 were detected to have lateral lymph node metastases within six months after the initial surgery. In total, 35 (16.4%) of 213 patients with CLNM in the no-TCI group were considered as having preoperative LLNM. For patients with positive CLNM, those within the TCI group showed a significantly higher LLNM rate than those within the no-TCI group (p-value=0.001). Moreover, 35 (3.5%) of all the 998 patients enrolled experienced tumor recurrence in our study, with 16 (2.6%) in the no-TCI group and 19 (5.1%) in the TCI group. Patients within the TCI group exhibited a significantly higher rate of tumor recurrence than those within the no-TCI group (p-value=0.039, **Table 1**).

### 3.2 Comparisons between patients with CLNM and LLNM or not for patients within no-TCI and TCI groups

First, we compared patients with CLNM or not within no-TCI and TCI groups. For patients within the no-TCI group, the presences of bilateral disease and multifocality were significantly more frequently detected in patients with positive CLNM than in those with negative CLNM (p-value=0.000 and 0.000, respectively, shown in **Supplementary Table S1**). 47.4% of patients are men within the positive CLNM group, the rate of which was significantly higher than that of patients with negative CLNM (28.4%, p-value=0.000). Moreover, patients with positive CLNM also showed younger ages and significantly larger tumor sizes than those with negative CLNM among the no-TCI group (p-value=0.000 and 0.000, respectively). For patients within the TCI group, patients with positive CLNM also showed younger ages, significantly larger tumor sizes, increased percentage of males, and more frequent detection of multifocality than those with negative CLNM (p-value<0.05). However, no significant difference was found in terms of the presence of bilateral disease between patients with CLNM or not among patients within the TCI group.

In terms of LLNM, 35 in 213 and 85 in 290 patients with positive CLNM showed LLNM in no-TCI and TCI groups respectively. For patients within no-TCI and TCI groups, patients with positive LLNM both showed significantly larger tumor sizes, large maximum positive CLN diameter (MCLND), more positive CLN numbers (CLNN), and more frequent detection of iNG than those with negative LLNM (p-value<0.05, **Supplementary Table S1**). For patients within the no-TCI group, the presence of bilateral disease was significantly more frequent in patients with positive LLNM than that in patients with negative LLNM (45.7% vs. 23.0%, p-value=0.006). For patients within the TCI group, no significant difference was found in terms of the presence of bilateral disease between patients with LLNM or not. However, an significantly increased percentage of males was found in patients exhibiting positive LLNM than in those without (51.8% vs. 40.0%, p-value=0.008).

### 3.3 Surgery-related complications after CLND alone and CLND+LLND

Among the 998 patients enrolled, 874 received CLND only and the other 124 received both CLND and LLND. Patients receiving CLND alone showed a comparable incidence rate of temporary postoperative hypoparathyroid hormone and hoarseness with those receiving LLND (20.8% vs. 23.4% and 10.1% vs. 12.9%, p-value=0.513 and 0.334, respectively, **Supplementary Table S2**). However, those receiving CLND+LLND showed significantly higher incidence rates of relatively severe postoperative complications including permanent postoperative hypoparathyroid hormone (4.8% vs. 1.6%, p-value=0.016) and hoarseness (4.0% vs. 1.5%, p-value=0.046), and chyle leakage (3.2% vs. 0.2%, p-value=0.000) than those receiving CLND alone.

### 3.4 Risk stratification for CLNM in patients within no-TCI and TCI groups

The central lymph node involvement rates for no-TCI and TCI groups were 34.2% (213 in 622) and 77.1% (290 in 376), respectively. Univariate and multivariate logistic regression analyses were then performed to screen out independent risk factors of CLNM for patients with negative and positive TCI. The results showed that factors including age less than 55 years old, male, the presence of bilateral disease and multifocality, and maximum tumor diameter (MTD)>=0.5cm were proven to be associated with CLNM for patients in the no-TCI group by univariate analysis, and were enrolled in the following multivariate analysis. The results showed that the aforementioned five factors were all confirmed to be independent risk factors of CLNM for patients with negative TCI (shown in **Table 2**).

**Table 2:** Univariate and multivariate analyses for PTC patients with negative TCI.

	Univariate analysis		Multivariate analysis			Univariate analysis		Multivariate analysis	
	Hazard ratio (95% CI)	P value	Hazard ratio (95% CI)	P value		Hazard ratio (95% CI)	P value	Hazard ratio (95% CI)	P value
*Analyzing all No-TCI patients to screen out independent factors for CLNM*					* Analyzing No-TCI patients with positive CLNM to screen out independent factors for LLNM*				
***Factors selected***					***Factors selected***				
**Age**		***0.002***		**0.001**	**Age**		0.517		
>= 55 vs. < 55	***0.505 (0.327-0.782)***		***0.428 (0.263-0.697)***		>= 55 vs. < 55	0.691 (0.226-2.112)			
**BMI**		0.780			**BMI**		0.436		
> 23 vs. <= 23	1.049 (0.752-1.462)				> 23 vs. <= 23	1.340 (0.641-2.802)			
**Gender**		***0.000***		***0.000***	**Gender**		0.881		
Male vs. Female	***2.278 (1.614-3.214)***		***2.267 (1.552-3.312)***		Male vs. Female	1.057 (0.512-2.183)			
**History of smoking**		0.071			**History of smoking**		0.230		
Yes vs. No	1.810 (0.950-3.446)				Yes vs. No	1.952 (0.655-5.823)			
**History of alcoholism**		0.207			**History of alcoholism**		0.414		
Yes vs. No	1.526 (0.792-2.940)				Yes vs. No	1.638 (0.501-5.354)			
**History of hypertension**		0.657			**History of hypertension**		0.162		
Yes vs. No	0.906 (0.585-1.402)				Yes vs. No	0.412 (0.119-1.427)			
**History of diabetes**		0.096			**History of diabetes**		0.447		
Yes vs. No	0.566 (0.290-1.106)				Yes vs. No	0.447 (0.056-3.574)			
**Bilateral disease**		***0.000***		***0.023***	**Bilateral disease**		***0.007***		***0.047***
Yes vs. No	***2.402 (1.583-3.645)***		***1.741 (1.081-2.803)***		Yes vs. No	***2.814 (1.328-5.963)***		***2.960 (1.016-8.620)***	
**Maximum tumor diameter**		***0.000***		***0.000***	**Maximum tumor diameter**		***0.030***		0.461
>= 0.5cm vs. < 0.5cm	***2.232 (1.584-3.147)***		***2.136 (1.463-3.117)***		>= 1.0cm vs. < 1.0cm	***2.281 (1.085-4.794)***		1.527 (0.496-4.699)	
**Tumor location**		0.464			**Tumor location**		0.438		
Upper vs. Middle/Lower	0.866 (0.589-1.274)				Upper vs. Middle/Lower	1.380 (0.612-3.113)			
**Multifocality**		***0.000***		***0.000***	**Multifocality**		***0.031***		0.956
Yes vs. No	***5.055 (3.438-7.434)***		***4.684 (3.095-7.089)***		Yes vs. No	***2.271 (1.076-4.795)***		1.032 (0.334-3.189)	
**PTC with ipsilateral nodular goiter**		0.857			**PTC with ipsilateral nodular goiter**		***0.011***		***0.045***
Yes vs. No	1.036 (0.707-1.517)				Yes vs. No	***2.673 (1.253-5.703)***		***3.066 (1.025-9.172)***	
**PTC with ipsilateral Hashimoto thyroiditis**		0.584			**PTC with ipsilateral Hashimoto thyroiditis**		0.788		
Yes vs. No	1.116 (0.754-1.652)				Yes vs. No	1.121 (0.487-2.579)			
					**Maximum diameter of positive CLN**		***0.000***		***0.000***
					> 1.0cm vs. <= 1.0cm	***34.172 (13.330-87.603)***		***27.588 (9.116-83.492)***	
					**Number of positive CLN**		***0.000***		0.199
					>=3 vs. <3	***2.827 (1.797-4.449)***		1.502 (0.807-2.794)	

PTC, papillary thyroid carcinoma; TCI, thyroid capsular invasion; CLNM, central lymph node metastasis; LLNM, lateral lymph node metastasis; BMI, body mass index; CLN, central lymph node. The bold values represent statistically significant.

For patients within the TCI group, factors including age less than 55 years old, male, history of diabetes, MTD>=1.0cm, and the presence of multifocality were proven to be associated with CLNM by univariate analysis and were then enrolled in multivariate analysis. As a result, all factors were confirmed to be independent risk factors of CLNM except the history of diabetes for patients with positive TCI (shown in **Table 3**).

**Table 3:** Univariate and multivariate analyses for PTC patients with positive TCI.

	Univariate analysis		Multivariate analysis			Univariate analysis		Multivariate analysis	
	Hazard ratio (95% CI)	P value	Hazard ratio (95% CI)	P value		Hazard ratio (95% CI)	P value	Hazard ratio (95% CI)	P value
*Analyzing all PTC patients with positive TCI to screen out independent factors for CLNM*					* Analyzing positive TCI patients with CLNM to screen out independent factors for LLNM*				
***Factors selected***					***Factors selected***				
**Age**		***0.000***		***0.000***	**Age**		0.785		
>= 55 vs. < 55	***0.266 (0.149-0.474)***		***0.286 (0.148-0.551)***		>= 55 vs. < 55	0.893 (0.397-2.011)			
**BMI**		0.380			**BMI**		0.578		
> 23 vs. <= 23	0.804 (0.494-1.308)				> 23 vs. <= 23	1.155 (0.695-1.919)			
**Gender**		***0.000***		***0.002***	**Gender**		***0.009***		0.507
Male vs. Female	***2.917 (1.614-5.271)***		***2.704 (1.432-5.107)***		Male vs. Female	***1.982 (1.187-3.311)***		1.283 (0.614-2.679)	
**History of smoking**		0.203			**History of smoking**		0.286		
Yes vs. No	2.019 (0.685-5.954)				Yes vs. No	1.575 (0.684-3.627)			
**History of alcoholism**		0.866			**History of alcoholism**		0.788		
Yes vs. No	0.926 (0.382-2.249)				Yes vs. No	1.137 (0.446-2.896)			
**History of hypertension**		0.118			**History of hypertension**		0.137		
Yes vs. No	0.629 (0.352-1.124)				Yes vs. No	0.568 (0.269-1.197)			
**History of diabetes**		***0.020***		0.105	**History of diabetes**		0.602		
Yes vs. No	***0.337 (0.135-0.843)***		0.397 (0.130-1.213)		Yes vs. No	1.397 (0.398-4.902)			
**Bilateral disease**		0.625			**Bilateral disease**		0.238		
Yes vs. No	1.151 (0.654-2.026)				Yes vs. No	1.403 (0.800-2.461)			
**Maximum tumor diameter**		***0.000***		***0.004***	**Maximum tumor diameter**		***0.000***		0.063
>= 1.0cm vs. < 1.0cm	***2.543 (1.513-4.274)***		***2.303 (1.315-4.032)***		>= 1.0cm vs. < 1.0cm	***2.958 (1.728-5.064)***		1.984 (0.964-4.081)	
**Tumor location**		0.564			**Tumor location**		0.086		
Upper vs. Middle/Lower	0.859 (0.512-1.440)				Upper vs. Middle/Lower	1.605 (0.935-2.755)			
**Multifocality**		***0.000***		***0.000***	**Multifocality**		0.134		
Yes vs. No	***4.007 (2.161-7.432)***		***4.159 (2.158-8.015)***		Yes vs. No	1.475 (0.887-2.452)			
**PTC with ipsilateral nodular goiter**		0.121			**PTC with ipsilateral nodular goiter**		***0.000***		***0.000***
Yes vs. No	0.677 (0.413-1.109)				Yes vs. No	***3.241 (1.907-5.507)***		***4.832 (2.303-10.137)***	
**PTC with ipsilateral Hashimoto thyroiditis**		0.612			**PTC with ipsilateral Hashimoto thyroiditis**		0.300		
Yes vs. No	0.847 (0.445-1.610)				Yes vs. No	0.671 (0.315-1.427)			
					**Maximum diameter of positive CLN**		***0.000***		***0.000***
					> 1.0cm vs. <= 1.0cm	***27.773 (13.703-56.291)***		***22.980 (10.473-50.420)***	
					**Number of positive CLN**		***0.000***		***0.033***
					>=3 vs. <3	***1.962 (1.445-2.663)***		***1.560 (1.036-2.350)***	

PTC, papillary thyroid carcinoma; TCI, thyroid capsular invasion; CLNM, central lymph node metastasis; LLNM, lateral lymph node metastasis; BMI, body mass index; CLN, central lymph node. The bold values represent statistically significant.

**Figure 2:**
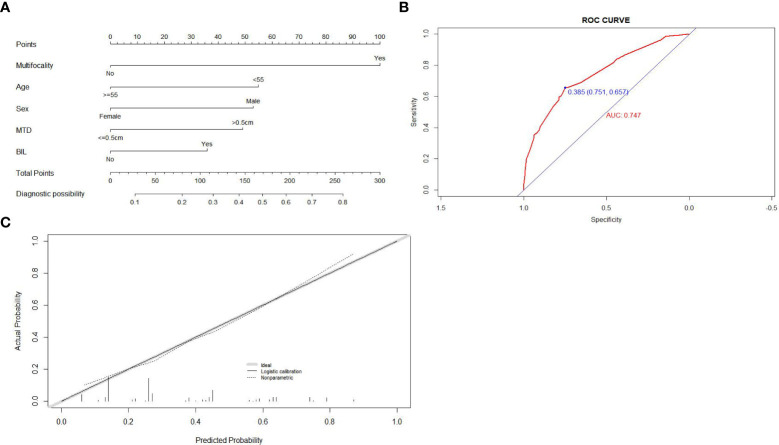
Construction, assessment, and validation of the predictive model of CLNM for patients within no-TCI group. **(A)** The nomograms for predicting CLNM risk in patients within no-TCI group; **(B)** The ROC curve and AUC of the nomogram for predicting CLNM risk in patients within no-TCI group; **(C)** The calibration curve of the nomogram for predicting CLNM risk in patients within no-TCI group. Actual probability is plotted on the y-axis, and nomogram predicted probability on the x-axis. CLNM, central lymph node metastasis; TCI, thyroid capsular invasion; ROC, receiver operating characteristics.

Then the five selected independent risk factors were used to create the prediction nomogram model of CLNM for patients with negative TCI (shown in **Figure 2A**). For evaluation and validation of the nomogram, we conducted an internal validation by 1000 bootstrap resamples to assess the prediction accuracy of CLNM for patients with negative TCI in terms of C-index. C-index turned out to be 0.747 (95% CI, 0.705-0.788), and 0.739 (95% CI, 0.724-0.754) after bootstrapping, which demonstrated good accuracy of our prediction model. The ROC curve and the calibration plot were shown in **Figures 2B, C**, both showing the fair agreement between the actual and assessed probability of CLNM in patients with negative TCI.

Each factor that made up the nomogram has its own risk points for CLNM. Then each patient within the no-TCI group would gain a total risk score by summing up the risk scores of the five selected factors in our nomogram. Based on the distribution of the total risk score, patients within the no-TCI group were classified into three subgroups by two cutoff values:

(1) a low CLNM risk subgroup (with CLNM risk score of <30, n=112),
(2) a moderate CLNM risk subgroup (30<= CLNM risk score of <80, n=194),
(3) a high CLNM risk subgroup (with CLNM risk score ≥80, n=316).

The CLNM rates of the low-risk subgroup were proven to be only 10.7% (12 in 112), while it went as high as 51.3% (162 in 316) for the high-risk subgroup, showing markedly different risks of CLNM among the three subgroups of patients in the no-TCI group (20.1% (39 in 194) for moderate-risk group, shown in **Supplementary Table S3**).

The CLNM rate of patients within the TCI group was up to 77.1% (290 in 376). Considering the extremely high central neck involvement rate, patients with positive TCI were all identified as having a high-risk of CLNM in our research.

### 3.5 Risk stratification for LLNM in patients within no-TCI group

**Figure 3:**
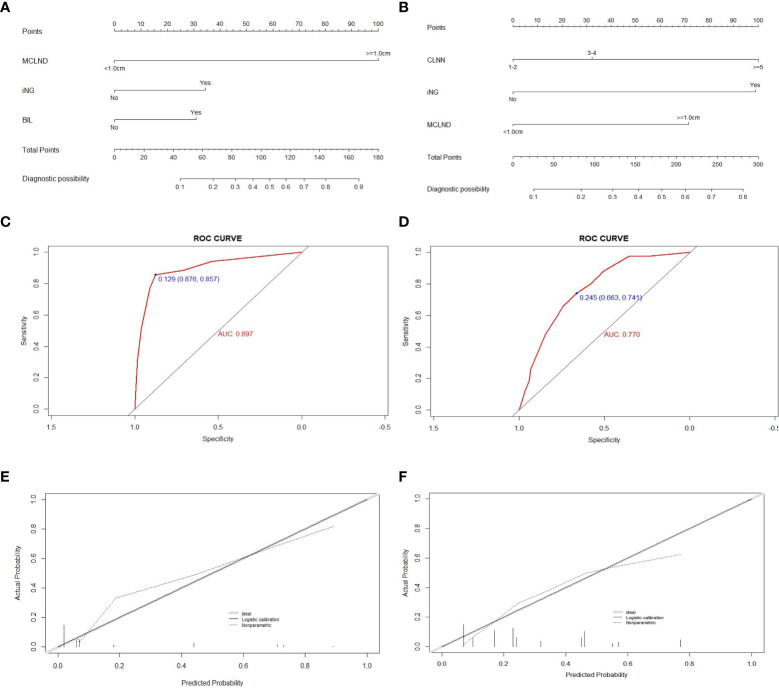
Construction, assessment, and validation of the predictive model of LLNM. **(A, B)** The nomograms for predicting LLNM risk in patients within no-TCI and TCI groups, respectively; **(C, D)** The ROC curve and AUC of the nomograms for predicting LLNM risk in patients within no-TCI and TCI groups, respectively; **(E, F)** The calibration curves of the nomogram for predicting LLNM risk in patients within no-TCI and TCI groups, respectively. Actual probability is plotted on the y-axis, and nomogram predicted probability on the x-axis. LLNM, lateral lymph node metastases; TCI, thyroid capsular invasion; ROC, receiver operating characteristics.

For the 213 patients with positive CLNM in the no-TCI group, univariate and multivariate analyses were conducted for screening out high-risk factors for LLNM and the results were shown in **Table 2**. Factors including the presence of bilateral disease and iNG, and MCLND>1.0cm were confirmed as independent factors for LLNM and were used for constructing a prediction model for patients with positive CLNM within the no-TCI group (**Figure 3A**). The C-index for predicting LLNM turned out to be 0.897 (95% CI, 0.833-0.961), and 0.881 (95% CI, 0.855-0.907) after bootstrapping, indicating satisfactory accuracy of our newly-established nomogram. The corresponding ROC curve and calibration plot were shown in **Figures 3C, E**, both showing great agreement between the actual and estimated possibility of LLNM in patients with positive CLNM in the no-TCI group.

According to the distribution of total scores based on the newly-created nomogram, patients with CLNM in the no-TCI group were also divided into three subgroups with extremely different lateral neck involvement rates: 1.0% (1 in 98), 9.7% (7 in 72), and 62.8% (27 in 43) for low-, moderate-, and high-risk subgroups (p-value=0.000, shown in **Supplementary Table S3**).

### 3.6 Risk stratification for LLNM in patients within TCI group

For the 290 patients with positive CLNM in TCI group, factors including MCLND>1.0cm, positive CLN number (CLNN)>=3, and the presence of iNG were confirmed as independent risk factors for LLNM (**Table 3**) and were used for constructing prediction model for patients with positive CLNM within no-TCI group (**Figure 3B**). The C-index was 0.770 (95% CI, 0.714-0.826), and 0.759 (95% CI, 0.739-0.779) after bootstrapping, exhibiting satisfactory accuracy. The corresponding ROC curve and calibration plot were exhibited in **Figures 3D, F**.

Paitents with positive CLNM in TCI group were also classified into three subgroups according to newly-created nomogram targeting them by two cutoff values:

(1) a low LLNM risk subgroup (with LLNM risk score of <50, n=75),
(2) a moderate LLNM risk subgroup (50<= LLNM risk score of <100, n=83),
(3) a high LLNM risk subgroup (with LLNM risk score ≥100, n=132).

The LLNM rates of low-, moderate-, and high-risk subgroups were proven to be 1.3% (1 in 75), 20.5% (17 in 83), and 50.8% (67 in 132) for low-, moderate-, and high-risk subgroups, indicating a significant different lateral neck involvement rates (p-value=0.000, shown in **Supplementary Table S3**).

### 3.7 Comprehensive cervical lymph node metastasis assessment flow chart for patients with PTC

**Figure 4:**
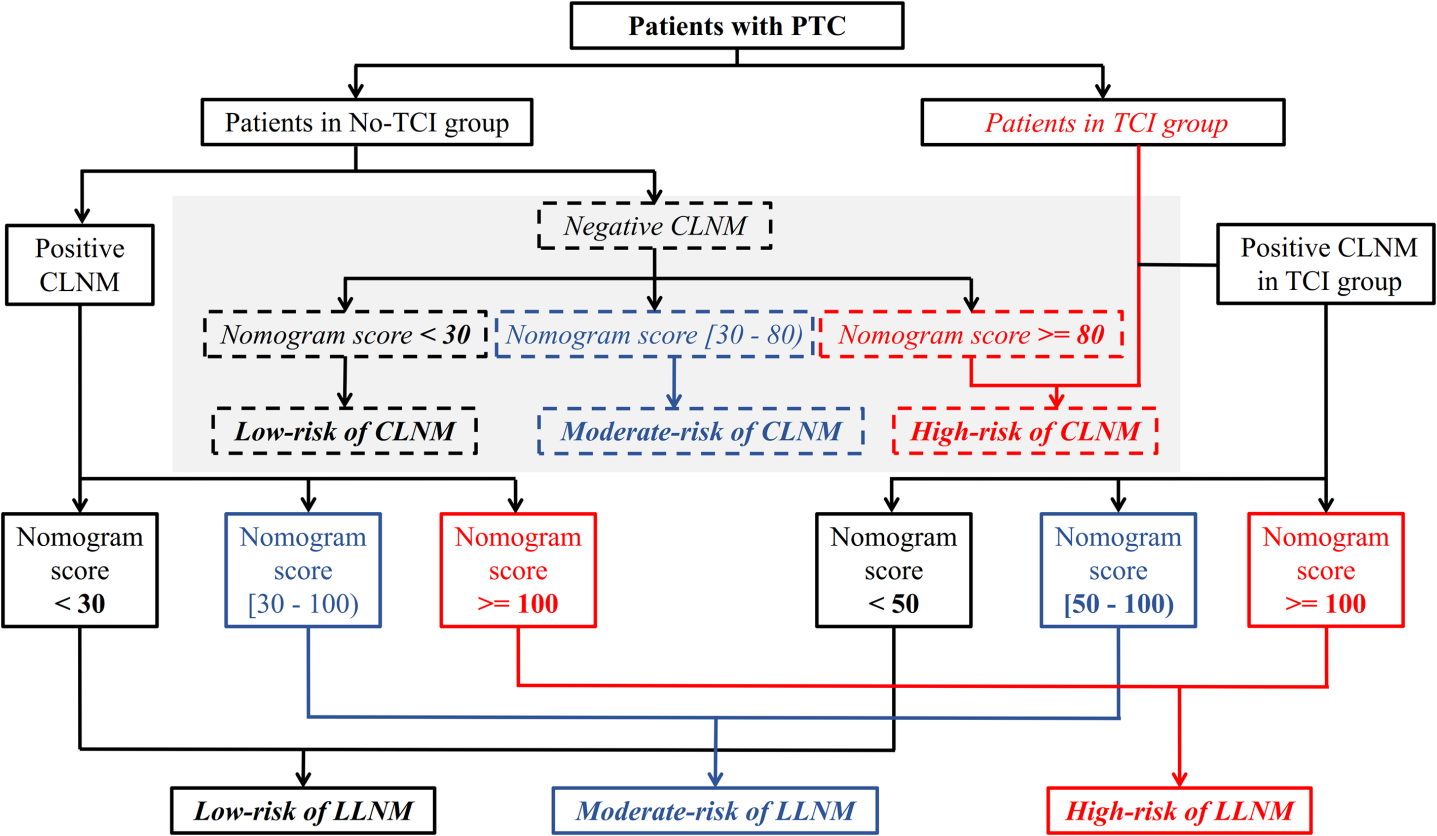
A meticulous and comprehensive stratification flow chart of cervical lymph node metastasis risk including both CLNM and LLNM for patients with PTC. CLNM, central lymph node metastasis; LLNM, lateral lymph node metastases; PTC, papillary thyroid carcinoma.

The aforementioned prediction models were integrated and were presented as a comprehensive cervical lymph node metastasis evaluation flow chart including both CLNM and LLNM for all patients with PTC in **Figure 4**.

## 4 Discussion

Debates on the optimal management strategy of neck regions for patients with PTC have existed for a long time. Many previous studies have screened out several risk factors for cervical lymph node metastasis including central and lateral neck involvement (8, 13, 15), for example, thyroid capsular invasion (TCI), larger tumor sizes, and younger ages for central lymph node metastasis (CLNM), and TCI, number of involved lymph nodes in central neck regions, and tumors located on upper portion for lateral lymph node metastasis (LLNM). Of these, capsular involvement is one of the most common and recognized risk factors for both CLNM and LLNM. Here in our research, based on whether TCI exists, patients with papillary thyroid carcinoma (PTC) were divided into two subgroups: TCI and no-TCI groups. The risk of CLNM and LLNM in each group was discussed and the risk prediction models of these two neck regions for the two groups were also constructed. Furthermore, we summarized and integrated the aforementioned prediction models, to establish a comprehensive evaluation process of cervical lymph node involvement risk for each patient with PTC, which provides certain guidance and basis for the standard clinical decision selection of cervical management for those patients.

TCI in our study was defined as a tumor clinging closely to the junction of thyroid and adjacent soft tissue, that can invade beyond the thyroid and surrounding tissues. Some previous studies take extrathyroidal extension (ETE) as the research index for predicting neck involvement for patients with PTC (16, 17), and those with microscopic and macroscopic ETE both showed significantly higher neck involvement rates and poor prognosis outcomes than those with negative ETE (18), indicating the pivotal role of capsular invasion status in the decision-making of management strategies for PTC patients. The reason why we chose TCI rather than ETE for patient stratification is because TCI is an extension of the concept of ETE, which includes both microscopic and macroscopic ETE, and also those where the tumor do not invade beyond the capsular and is merely in contact. Studies have shown that the probability of cervical lymph node metastasis in patients with positive TCI was significantly higher than in those with encapsulated tumors (8, 13, 19). Our results also confirmed the significantly high risk of both CLNM and LLNM for patients within the TCI group than the no-TCI group.

In terms of CLNM, younger age, male, the presence of multifocality, and large tumor size were confirmed to be independent factors for patients within both no-TCI and TCI groups, while the presence of bilateral disease was identified to be closely associated with CLNM only for patients within the no-TCI group. The CLNM rate was 34.2% (213 in 622) for all patients within the no-TCI group, and 10.7% (12 in 112) and 51.3% (162 in 316) for low- and high- CLNM risk subgroups after stratification using our newly-created nomogram, demonstrating excellent results for screening out patients with high risks of CLNM within this population that was traditionally considered to be low-risk (20). However, for patients with positive TCI, the CLNM rate was up to nearly 80%, indicating the extremely high risk of CLNM in those patients.

As for lateral neck metastasis, considering that skip metastasis (positive LLNM with no CLNM involvement) is rare in PTC patients, the risk of LLNM was analyzed for those with positive CLNM in no-TCI and TCI groups respectively. Features including both primary tumor and metastatic lymph nodes of the central compartment were enrolled to select out risk factors of LLNM for patients within different groups. Although factors including the presence of iNG and larger positive CLN size were proven to be independent risk factors of lateral neck involvement for both patients within no-TCI and TCI groups, the difference still exists between these two groups: the presence of bilateral disease was significantly associated with LLNM in patients of the no-TCI group rather than TCI group, while higher count of positive CLN was identified as high-risk factors for patients within TCI group, which further indicates the significant implications of our study to separate patients by the presence of TCI. Predictive nomograms were established for patients within these two groups based on their respective risk factors. As a result, although the presence of positive CLNM was repeatedly proven by previous studies as a high-risk factor of LLNM (13, 15, 21, 22) for patients with PTC, here in our study, a small portion of patients with total LLNM risk scores <30 and <50 according to their own predictive nomograms were screened out and were considered as low-risk subgroup of LLNM within these patients, with LLNM rate of 1.0% (1 in 98) and 1.3% (1 in 75) for patients within no-TCI and TCI groups, respectively.

Finally, all the aforementioned results were integrated as a detailed cervical metastasis risk stratification flow chart for patients with PTC. Even though no clinically positive CLNM was found, patients with positive TCI, and patients who show negative TCI and with a total score no less than 80 based on the corresponding nomogram, are regarded as having a high risk of CLNM, and a closer follow-up scheme should be conducted for them. Considering that the incidence rates of postoperative complications including permanent postoperative hypoparathyroid hormone and hoarseness, and chyle leakage were proven to be significantly higher in those receiving lymph node dissection involving lateral neck regions in our study, the administration of prophylactic LLND should be minimized for PTC patients. Using the newly-created risk stratification flow chart for LLNM in our study, for patients with clinically positive CLNM, those with positive and negative TCI and with a total score no less than 100 according to their respective prediction models, are identified as having a high risk of LLNM, and a closer examination of lateral neck regions as well as a follow-up with shorter intervals should be conducted for these patients. Even LLND with a prophylactic purpose could be considered based on patients’ preferences. However, no intervention of the lateral neck region is needed for patients classified as low-risk of LLNM (patients within no-TCI and TCI groups who received a total score of less than 30 and 50 based on their respective prediction models) considering the extremely low lateral neck involvement rates.

## 5 Conclusions

A meticulous and comprehensive stratification flow chart for PTC patients for quantitatively evaluating cervical lymph node metastasis risk including both CLNM and LLNM was constructed, which may aid in clinical decision-making for the management of neck regions.

## Data availability statement

The raw data supporting the conclusions of this article will be made available by the authors, without undue reservation.

## Ethics statement

The studies involving human participants were reviewed and approved by the Institutional Ethics Committee of the Eye & ENT Hospital of Fudan University, the Ruijin Hospital of Shanghai Jiao Tong University School of Medicine, and the Department of General Surgery, Civil Aviation Shanghai Hospital. The patients/participants provided their written informed consent to participate in this study.

## Author contributions

(1) Conception and design or analysis and interpretation of data: all authors. (2) Drafting of the manuscript or revising it for important intellectual content: ZY, YH, LT. (3) All authors contributed to the article and approved the submitted version.
